# Efficacy of total hip arthroplasty for the treatment of patients with femoral head avascular necrosis: A protocol for systematic review and meta-analysis

**DOI:** 10.1097/MD.0000000000020259

**Published:** 2020-05-15

**Authors:** Xiao-feng Qiao, Yu Xue, Shi-chen Liu, Qing-hui Ji

**Affiliations:** a First Ward of Orthopedis Department, First Affiliated Hospital of Jiamusi University; b Department of Obstetrics and Gynecology, Second Affiliated Hospital of Jiamusi University, Jiamusi, China.

**Keywords:** complications, efficacy, femoral head avascular necrosis, total hip arthroplasty

## Abstract

**Background:**

Femoral head avascular necrosis (FHAN) is a very common condition among elderly population. Previous studies have reported that total hip arthroplasty (THAP) can benefit patients with such condition. However, no study systematically addressed this topic. Thus, this study will systematically explore the efficacy and safety of THAP for the treatment of patients with FHAN.

**Methods:**

We will search the following electronic bibliographic databases from inception to the February 29, 2020: MEDLINE, EMBASE, Cochrane Library, Cumulative Index to Nursing and Allied Health Literature, China National Knowledge Infrastructure, and Chinese Scientific Journal Database. Randomized controlled trials of THAP for the treatment of patients with FHAN will be included, which were reported in any language. All process of study selection, data collection, and study quality assessment will be performed independently by 2 authors independently. Any divergences will be solved by discussion with another author. RevMan 5.3 software will be carried out for data synthesis and analysis.

**Results:**

This study will provide a detailed summary of current evidence related to the efficacy and safety of THAP for the treatment of patients with FHAN through assessing pain intensity of hip or knee joints, function and limitation of attacked femoral head, health-related quality of life, and complications.

**Conclusion:**

The findings of this study may provide helpful guidance of THAP for the treatment of patients with FHAN.

**Systematic review registration:**

INPLASY202040067.

## 1 Introduction

Femoral head avascular necrosis (FHAN) is a very common and complicated disorder in osteonecrosis.^[1,2,3,4,5]^ It occurs because of the disruption of blood supply to the head of femur.^[6,7,8,9,10]^ It has been estimated about 10000 to 20000 new cases reported annually in the USA.^[11]^ Although current interventions have reported to treat FHAN, there is still limited efficacy and thus further cause damage of femoral head.^[12,13,14,15,16,17]^ Fortunately, total hip arthroplasty (THAP) have reported to treat FHAN effectively.^[18,19,20,21,22,23]^ However, no systematic review has been undertaken to assess the efficacy and safety of THAP for FHAN. Therefore, this study will assess the efficacy and safety of THAP for the treatment of patients with FHAN.

## 2 Methods and analysis

### 2.1 Study registration

We have registered this protocol on INPLASY202040067. We report it following the guideline of Preferred Reporting Items for Systematic Reviews and Meta-Analysis Protocol statement guidelines.^[24,25]^

### 2.2 Eligibility criteria for study selection

#### 2.2.1 Types of study

Randomized controlled trials (RCTs) of THAP for the treatment of patients with FHAN, which were reported in any language, will be included. Any uncontrolled trial, non-RCTs and quasi-RCTs will all be excluded.

#### 2.2.2 Types of participant

Participants who were diagnosed as FHAN will be included regardless their age, sex, and source of studies.

#### 2.2.3 Types of intervention

##### 2.2.3.1 Interventions

Any types of THAP therapy used in patients as interventional intervention will be included in this study.

##### 2.2.3.2 Comparators

We will include patients who received any management as a control intervention in this study. We will exclude any combined therapy with THAP as their comparators.

#### 2.2.4 Types of outcome measurement

Primary outcome includes pain intensity. It is measured by any validated pain scales, such as Numerical Rating Scale.

Secondary outcomes are function and limitation of attacked femoral head (as checked by any relevant validated indexes, including Western Ontario and McMaster Universities Osteoarthritis Index), health-related quality of life (as evaluated by any related tools, such as 36-Item Short Form Health Survey), and any complications post surgery.

### 2.3 Literature search

The following electronic bibliographic databases will be searched from inception to the February 29, 2020: MEDLINE, EMBASE, Cochrane Library, Cumulative Index to Nursing and Allied Health Literature, China National Knowledge Infrastructure, and Chinese Scientific Journal Database. We will identify any potential RCTs of THAP for the treatment of patients with FHAN. We will search all these electronic databases without any language and publication status restrictions. The search strategy for MEDLINE is presented (Table 1). We will modify similar search strategies to all other electronic databases.

**Table 1:**
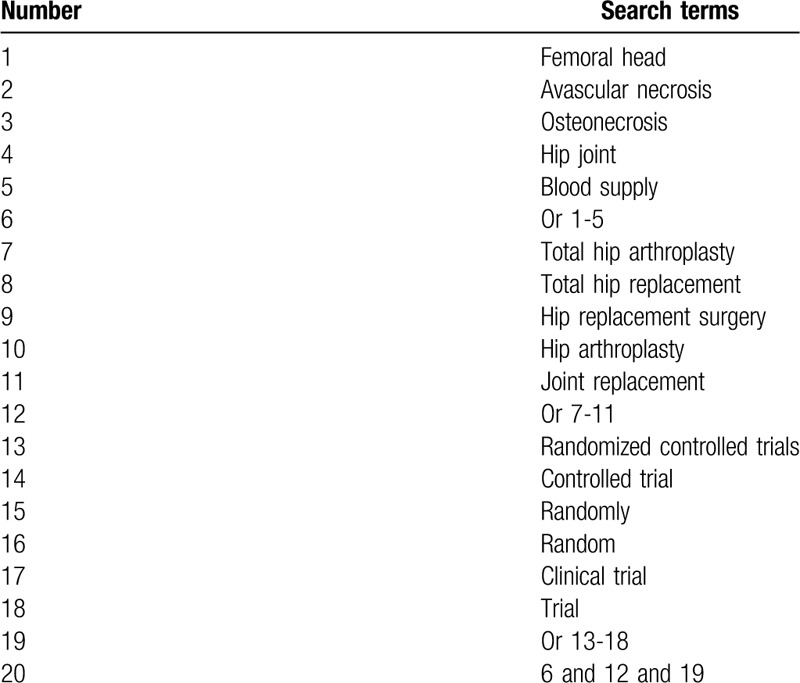
Search strategy of MEDLINE.

Other resources for potential studies are also searched, such as websites of clinical trial registry, and reference lists of included trials and relevant reviews.

### 2.4 Study selection

The titles/abstracts of all retrieved literatures will be scanned independently by 2 authors based on the predefined eligibility criteria. All unconnected studies will be removed. If necessary, full text of remaining potential trials will be read cautiously against all inclusion criteria. Any different views on the study selection will be arbitrated by a third author. All excluded literatures will be noted in a table with specific reasons for their exclusion. The study selection process is exerted in a flowchart.

### 2.5 Data extraction and management

The following data will be extracted from all included RCTs by 2 independent authors using predefined data acquisition form: reference ID, author information, publication time, patient characteristics, sample size, study setting, study methods, details of intervention and controls, outcome indicators at all reported time points, complications, follow-up information, results, findings, and conflict of interests. Any different opinions will be arbitrated by a third author through discussion.

### 2.6 Missing data dealing with

If we identify any missing or unclear data, we will connect corresponding authors or relevant authors to obtain it. We will use an intention-to-treat analysis in case of missing data if possible. The potential impacts of missing data will be discussed as a limitation in this study.

### 2.7 Risk of bias assessment

Risk of bias for each eligible trial will be assessed by 2 independent authors using Cochrane Risk of Bias Tool. Two authors will independently judge them for each study on 7 aspects. Each aspect will be categorized as high, unclear or low risk of bias. Any disagreements regarding the risk of bias assessment will be solved by a third author through discussion.

### 2.8 Statistical analysis

RevMan 5.3 software will be used for data synthesis and statistical analysis.

#### 2.8.1 Treatment effect measurement

As for dichotomous outcome data (such as incidence of complications), risk ratio and 95% confidence intervals will be employed. As for continuous outcome data (such as pain intensity of hip or knee joints), mean difference or standardized mean difference and 95% confidence intervals will be exerted.

#### 2.8.2 Heterogeneity assessment

Statistical heterogeneity will be investigated using *I*^2^ test. *I*^2^ ≤ 50% means little or no statistical heterogeneity across the included trials, and a fixed-effects model will be practiced. *I*^2^ >50% indicates considerable heterogeneity, and a random-effects model will be utilized.

#### 2.8.3 Data synthesis

If statistical heterogeneity is minor among included trials, we will undertake meta-analysis based on the similar study and patient characteristics, interventions, controls, and outcome indicators. If statistical heterogeneity is considerable across the eligible studies, we will perform subgroup analysis to explore possible sources for such heterogeneity. If it is still not possible to conduct meta-analysis after subgroup analysis, we will report outcome results as a narrative summary.

#### 2.8.4 Subgroup analysis

If data are available, we will carry out subgroup analysis to explore the sources of considerable heterogeneity based on the variations in study characteristics, different types of interventions and controls, and outcomes.

#### 2.8.5 Sensitivity analysis

We will conduct a sensitivity analysis to monitor the robustness of the study findings based on the methodological weaknesses and missing data.

#### 2.8.6 Publication bias

Funnel plot and Egger test will be generated to observe any potential publication biases when at least 10 eligible trials are included in this study.

#### 2.8.7 Summary of evidence

Two authors will independently appraise the quality of evidence for main outcome indicators using the Grading of Recommendations Assessment, Development, and Evaluation System approach.^[26,27]^ Its results will be demonstrated in the ‘summary of findings’ tables in the final report. Any different views will be solved by a third author through discussion.

### 2.9 Dissemination and ethics

This study dose not requires ethical approval, because it will not use individual patient data. This study is expected to be published through a peer-reviewed journal.

## 3 Discussion

This study firstly investigated the efficacy and complications of THAP for the treatment of patients with FHAN. The findings of this study will provide a detailed and summary of the existing evidence relevant of THAP in pain relief of knees and hips, function improvements of attacked joints, and health-related quality of life in patients with FHAN.

Moreover, it may also provide helpful reference and recommendation for clinicians and further researches.

## Acknowledgment

This study was supported by Heilongjiang Provincial Universities Basic Research Business Expenses Research Project (2017-KYYWF-0574). The supporter did not involve in the whole process of this study.

## Author contributions

**Conceptualization:** Xiao-feng Qiao, Qing-hui Ji.

**Data curation:** Xiao-feng Qiao, Yu Xue, Shi-chen Liu, Qing-hui Ji.

**Formal analysis:** Yu Xue.

**Funding acquisition:** Qing-hui Ji.

**Investigation:** Qing-hui Ji.

**Methodology:** Xiao-feng Qiao, Yu Xue, Shi-chen Liu.

**Project administration:** Qing-hui Ji.

**Resources:** Xiao-feng Qiao, Yu Xue, Shi-chen Liu.

**Software:** Xiao-feng Qiao, Yu Xue, Shi-chen Liu.

**Supervision:** Qing-hui Ji.

**Validation:** Xiao-feng Qiao, Yu Xue, Shi-chen Liu, Qing-hui Ji.

**Visualization:** Yu Xue, Shi-chen Liu, Qing-hui Ji.

**Writing – original draft:** Xiao-feng Qiao, Yu Xue, Shi-chen Liu, Qing-hui Ji.

**Writing – review and editing:** Xiao-feng Qiao, Shi-chen Liu, Qing-hui Ji.
